# Impact of "test and treat" recommendations on eligibility for antiretroviral treatment: Cross sectional population survey data from three high HIV prevalence countries

**DOI:** 10.1371/journal.pone.0207656

**Published:** 2018-11-26

**Authors:** Menard Laurent Chihana, Helena Huerga, Gilles Van Cutsem, Tom Ellman, Stephen Wanjala, Charles Masiku, Elisabeth Szumilin, Jean Francois Etard, Mary-Ann Davies, David Maman

**Affiliations:** 1 Centre for Infectious Diseases and Epidemiology, University of Cape Town, Cape Town, South Africa; 2 Epicentre, Cape Town, South Africa; 3 Epicentre, Paris, France; 4 Southern Africa Medical Unit, Médecins Sans Frontières, Cape Town, South Africa; 5 Médecins Sans Frontières, Nairobi, Kenya; 6 Médecins Sans Frontières, Lilongwe, Malawi; 7 Médecins Sans Frontières, Paris, France; 8 IRD UMI 233, INSERM U1175, Montpellier University, TransVIHMI, Montpellier, France; NPMS-HHC CIC / LSH&TM, UNITED KINGDOM

## Abstract

**Background:**

Latest WHO guidelines recommend starting HIV-positive individuals on antiretroviral therapy treatment (ART) regardless of CD4 count. We assessed additional impact of adopting new WHO guidelines.

**Methods:**

We used data of individuals aged 15–59 years from three HIV population surveys conducted in 2012 (Kenya) and 2013 (Malawi and South Africa). Individuals were interviewed at home followed by rapid HIV and CD4 testing if tested HIV-positive. HIV-positive individuals were classified as “eligible for ART” if (i) had ever been initiated on ART or (ii) were not yet on ART but met the criteria for starting ART based on country’s guidelines at the time of the survey (Kenya–CD4< = 350 cells/μl and WHO Stage 3 or 4 disease, Malawi as for Kenya plus lifelong ART for all pregnant and breastfeeding women, South Africa as for Kenya plus ART for pregnant and breastfeeding women until cessation of breastfeeding).

**Findings:**

Of 18,991 individuals who tested, 4,113 (21.7%) were HIV-positive. Using country’s ART eligibility guidelines at the time of the survey, the proportion of HIV-infected individuals eligible for ART was 60.0% (95% CI: 57.2–62.7) (Kenya), 73.4% (70.8–75.8) (South Africa) and 80.1% (77.3–82.6) (Malawi). Applying WHO 2013 guidelines (eligibility at CD4< = 500 and Option B+ for pregnant and breastfeeding women), the proportions eligible were 82.0% (79.8–84.1) (Kenya), 83.7% (81.5–85.6) (South Africa) and 87.6% (85.0–89.8) (Malawi). Adopting “test and treat” would mean a further 18.0% HIV-positive individuals (Kenya), 16.3% (South Africa) and 12.4% (Malawi) would become eligible. In all countries, about 20% of adolescents (aged 15–19 years), became eligible for ART moving from WHO 2013 to “test and treat” while no differences by sex were observed.

**Conclusion:**

Countries that have already implemented 2013 WHO recommendations, the burden of implementing “test and treat” would be small. Youth friendly programmes to help adolescents access and adhere to treatment will be needed.

## Introduction

HIV remains one of the biggest contributors to mortality and morbidity in the world with most deaths occurring in the Sub-Saharan Africa (SSA)[1]. Despite freely available treatment for HIV/AIDS over the past decade, only (66.0%) of people living with HIV in eastern and southern part of SSA were on treatment in 2017 [2]. The World Health Organisation (WHO) 2013 treatment guidelines for starting HIV-positive people on antiretroviral therapy (ART) were CD4< = 500 cells/μl and for pregnant women to commence ART regardless of CD4 cell count [3]. In 2015 the WHO guidelines changed to starting every HIV-positive person on ART regardless of CD4 cell count[4] (the so-called “test and treat” approach) although some countries have not yet implemented these recommendations.

With only half the population of HIV-positive individuals on treatment, containing the spread of HIV remains a challenge with only small declines in incidence[5]. However, more evidence is becoming available on the benefits of undetectable viral load and early ART initiation on mortality and morbidity[6–8] and on lowering the risk of transmission[9–13], bringing hope on how further spreading of the disease can be contained through a “test and treat” approach.

However, adopting the new WHO guidelines may have challenges such as costs associated with more people on ART, infrastructure, human resources and how to monitor everyone started on ART to ensure that they adhere to medication[4]. This makes it difficult for countries to transition if they do not know what to expect if they move to test and treat.

To plan properly for transitioning to the new WHO guidelines, countries need to know the number, proportion, age and sex distribution of the additional HIV-positive individuals that will need to start ART. Most studies on the impact of change in ART guidelines on eligibility have been based on mathematical modeling [14, 15] which can easily over or underestimate results depending on the model assumptions. Other studies however, have used population data, for example in Kenya, a study that estimated the impact of change in treatment guidelines using nationally representative Kenya AIDS indicator survey data fell short of measuring the differential impact of the new WHO guidelines on age and sex[16]. Our aim therefore, was to measure the impact of a “test and treat” policy on eligibility, stratified by sex and age, using population data from three countries (Kenya, Malawi and South Africa) at different stages of implementing previous WHO guidelines.

## Methods

### Study design

We used data from three HIV Impact in Population Surveys; Chiradzulu (Malawi), Ndhiwa (Kenya) and Eshowe (South Africa). These were population representative household based surveys and used largely similar sampling design and data collection methods, which are explained in detail elsewhere [17–19]. Briefly, a two stage sampling method was used to randomly select households for interviews. First, clusters were selected with a probability of clusters to be selected proportional to the number of households in the cluster (Kenya and Malawi) while for South Africa, the clusters were selected with probability proportion to population aged 15–59 making it a self-weighting sample. All three surveys used the respective country National census enumeration areas to define clusters. After first stage systematic sampling of clusters, from each cluster, households were selected using simple random selection (25 households per cluster for Malawi and South Africa, and 20 per cluster for Kenya). Households where no individual was found to provide information on the day of household visit or on two subsequent visits were replaced by another household on reserve selected using the same method. Adults aged between 15 and 59 years old residents and the visitors who had spent the previous night in the selected household were eligible for inclusion in the study.

### Data collection methods

The studies were conducted between September and November 2012 (Kenya), February and May 2013 (Malawi) and July and October 2013 (South Africa). For eligible members who agreed to participate in the survey, face to face individual interviews were conducted at a private place of the household using a structured survey questionnaire which included questions on knowledge of HIV/AIDS, ART use for HIV-positive individuals only, (self-reported in Kenya and Malawi, blood tested in South Africa) and pregnancy and reproduction for women.

For those who consented for HIV testing, (parental/guardian consent was sought first from minor’s guardian younger than 18 in Kenya and South Africa but parental consent was not required in Malawi where minors between 14–17 are considered mature to give own consent for HIV test) rapid HIV test and counseling was administered. Determine Rapid HIV-1/2 Antibody test (Abbott Laboratories, Abbott Park, IL, USA) was used for screening with Unigold Rapid HIV Test (Trinity Biotech PLC, Bray, Ireland) to confirm positive screening results. In case of discordant results, an ELISA test (Genetic Systems HIV-1/HIV-2 Plus O EIA, Bio-Rad, Redmond, WA, USA) was used to confirm infection. A venous blood sample was collected from all individuals who tested HIV-positive for CD4 measurement using home-based (PIMA CD4 counter, Alere PIMA, Jena, Germany) (Malawi and Kenya) or laboratory-based based FACSCalibur device (Becton, Dickinson and Company (BD)) (for South Africa). Further laboratory tests were Viral load testing (G2 real-time PCR, Biocentric, Bandol, France) (Kenya and Malawi); (NucliSens EasyQ HIV-1 v2.0 assay from Biomerieux) (South Africa) and presence of antiretroviral drugs in the blood.

### Statistical methods and analysis

Data were analyzed using Stata 13 (Stata Corporation, College Station, Texas). We used Chi-squared tests of association to check the distribution of demographic characteristics by sex in the population. We compared the proportion of individuals eligible for ART according to guidelines that were being used at the time of the survey in the relevant country with the proportion of individuals who would become eligible if the country were to switch to WHO 2015 guidelines. Individuals in need of ART were defined based on the relevant National ART Guidelines implemented at the time of the survey. For all the three countries, HIV-positive adults were eligible for ART if their CD4 cell count was < = 350 cells/μl, however prevention of mother to child transmission (PMTCT) guidelines differed by country. Kenya was using “Option A” guidelines where HIV-positive pregnant and breastfeeding women were started on ART if CD4 count was < = 350 cells/μl [20], Malawi was using “Option B+” i.e. lifelong ART for all HIV-positive pregnant and breastfeeding women irrespective of CD4 count [21] while South Africa was using “Option B” i.e. ART for all HIV-positive pregnant and breastfeeding women until cessation of breastfeeding [22]. In this cross-sectional analysis, among patients already on ART, we were unable to assess their historical eligibility at the time of ART initiation as we did not have access to previous laboratory or clinical records. For the purpose of this analysis, we classified HIV-positive individuals as “eligible for ART” if they either (i) had ever been initiated on ART before (according to self-report (Kenya and Malawi) and laboratory tested (South Africa)) or (ii) were not yet on ART but met the criteria for starting ART based on the guidelines for their country at the time. Malawi implemented WHO 2013 guidelines which we shall refer to as “CD4< = 500” in 2014 and implemented WHO 2015 guidelines which were shall call “test and treat” in April 2016; South Africa implemented “CD4< = 500” in January 2015 and “test and treat” in September 2016. Kenya implemented “test and treat” in July 2016.

The analysis was stratified by sex and age (grouped into 4 different categories, 15–19, 20–34, 35–44 and 45–59 years old).

### Ethics

All three surveys received ethical approval from both local and international ethics committee. Local approvals were obtained from the Kenya Medical Research Institute Ethical Review Committee (KEMRI, protocol number 347) (Kenya), National Health Sciences Research Committee (protocol number 1085) (Malawi) and the University of Cape Town Human Research Ethics Committee (HREC) protocol number 461/2012, and the Health Research Committee of the Health Research and Knowledge Management Unit of Kwazulu-Natal Department of Health (South Africa). All surveys received international approval from the Comite´ de Protection des Personnes d’Ile de France (protocol number 12056 for Kenya, 12084 for Malawi and 12091 for South Africa). All study participants in all the three surveys were asked to sign a written consent if they accepted to be included in the study.

## Results

**Fig 1:**
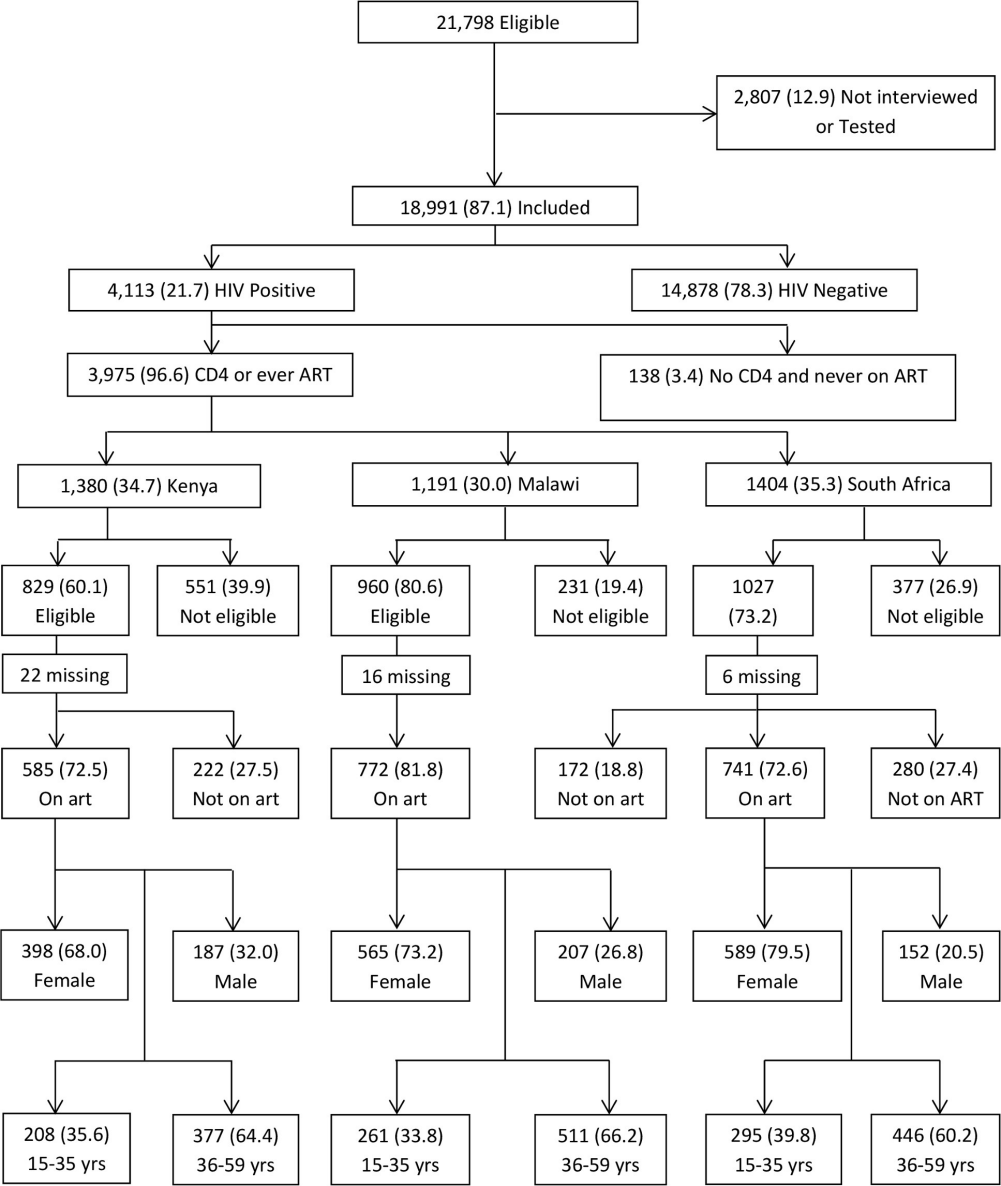
Flow chart showing individuals included in the survey. Distribution of individuals included in the survey by HIV status, country, ART eligibility at the time of the survey, ART status, sex and age.

A total of 18,991 (87.1%) out of 21,798 eligible individuals were included in the surveys, 1,403 (6.4%) (74.8% male) were absent from home, 1084 (5.0%) (53.5% male) refused to be interviewed or refused HIV test, 169 (0.8%) were incapacitated and 151 (0.7%) had other reasons for non-participation Fig 1. Among those included in the surveys, 6,076 (38.2% male) were from Kenya, 7,269 (41.2% male) from Malawi and 5,643 (37.7% male) were from South Africa.

In total, 4,113 (21.7%) participants were HIV-positive. HIV prevalence was highest in South Africa 25.2% (95% Confidence Interval [CI]: 23.6–26.9) followed by Kenya 24.1% (22.7–25.6) and Malawi 17.0% (16.0–18.1). In all three countries, females had a much higher prevalence than males; this difference was greatest in South Africa with prevalence among women almost twice as high as men 30.9% (29.0–32.9) compared to 15.9% (14.0–18.0).

Table 1 shows the distribution of baseline characteristics among all participants by sex. In all three countries, the proportion of adolescents (aged 15–19 years) was higher among men than among women. In Kenya and Malawi, more than 50% of the women were still breastfeeding while only 31.2% were still breastfeeding in South Africa and only less than 10% in all three countries were pregnant at the time of the survey. Among HIV-positive individuals, CD4 test results were available for 3,957 (96.2%) individuals. In all three countries, HIV positivity rate was higher among older men than women and there were more women with higher CD4 cell count of > = 500 cells/μl than men Table 2.

**Table 1:** Showing distribution of social-demographic characteristics of 18,991 participants by sex.

**Country**	**Kenya**				**Malawi**				**South Africa**			
**Variable**	**N(%)**	**Male n (%)**	**Female n(%)**	**P value**	**N(%)**	**Male n(%)**	**Female n(%)**	**P value**	**N(%)**	**Male n(%)**	**Female n(%)**	**P value**
Overall	6076 (100)	2321 (100)	3755 (100)	-	7269 (100)	2995 (100)	4274 (100)	-	5646 (100)	2131 (100)	3515(100)	-
**Age group**												
15–19	1260 (20.7)	579 (25.0)	681 (18.1)	<0.001	1563 (21.5)	785 (26.2)	778 (18.2)	<0.001	1452 (25.7)	679 (31.9)	773 (22.0)	<0.001
20–34	2583 (42.5)	880 (37.9)	1703 (45.4)		3165 (43.5)	1200 (40.1)	1965 (46.0)		2335 (41.4)	911 (42.8)	1424 (40.5)	
35–44	1097 (18.1)	399 (17.2)	698 (18.6)		1392 (19.2)	570 (19.0)	822 (19.2)		785 (13.9)	251 (11.8)	534 (15.2)	
45–59	1136 (18.7)	463 (20.0)	673 (17.9)		1149 (15.8)	440 (14.7)	709 (16.6)		1074 (19.0)	290 (13.6)	784 (22.3)	
**Education**												
Primary or less	5046 (83.1)	1764 (76.1)	3282 (87.4)	<0.001	5444 (74.9)	2024 (67.7)	3420 (80.1)	<0.001	2841 (50.3)	1075 (50.5)	1766 (50.2)	0.87
Sec. or more	1026 (16.9)	555(23.9)	471 (12.6)		1820 (25.0)	968 (32.4)	852 (19.9)		2802 (49.7)	1055 (49.5)	1749(49.8)	
Missing	4(0.1)	2	2		5 (0.1)	3	2		1 (0.02)	0	1	
**Marital status**												
Never married	1291 (21.3)	773 (33.5)	518 (13.9)	<0.001	1703 (23.5)	1061 (35.6)	642 (15.1)	<0.001	4232 (75.0)	1786 (83.9)	2446 (69.6)	<0.001
Married/living with partner	4133 (68.0)	1474 (63.9)	2659 (71.2)		4648 (63.9)	1822 (61.1)	2826 (66.4)		1198 (21.2)	294 (13.8)	904 (25.7)	
Widowed	511 (8.4)	18 (0.8)	493 (13.2)		262 (3.6)	11 (0.4)	251 (5.9)		107 (1.9)	10 (0.5)	97 (2.8)	
Divorced/ Separated	105 (1.7)	42 (1.8)	63 (1.7)		626 (8.6)	89 (3.0)	537 (12.6)		104 (1.8)	39 (1.8)	65 (1.9)	
Missing	36 (0.6)	14	22		30 (0.4)	12	19		5(0.1)	3	2	
**Occupation**												
Farming	3911 (64.4)	1304 (56.2)	2619 (69.3)	<0.001	3282 (45.2)	1219 (40.7)	2063 (48.1)	<0.001	317 (5.6)	141 (6.6)	176 (5.0)	<0.001
Salaried employment	803 (13.2)	369 (16.0)	437(11.6)		1803 (24.8)	916 (30.8)	887 (20.8)		375 (6.6)	164 (7.7)	211 (6.0)	
Student/None	1288 (21.2)	608 (26.2)	688(18.2)		2136 (29.4)	831 (27.8)	1305 (30.7)		4689 (83.1)	1675 (78.6)	3014 (85.8)	
Other	67 (1.1)	38 (1.6)	29(0.8)		31 (0.4)	21 (0.7)	10 (0.2)		265 (4.7)	151 (7.1)	114 (3.2)	
Missing	7 (0.1)	2	5		17 (0.2)	8	9		0			
**Country**	**Kenya**				**Malawi**				**South Africa**			
**Variable**	**N(%)**	**Male n(%)**	**Female n(%)**	**P value**	**N(%)**	**Male n(%)**	**Female n(%)**	**P value**	**N(%)**	**Male n(%)**	**Female n(%)**	**P value**
**Residency status**												
Resident	5991 (98.6)	2305 (99.3)	3686 (98.2)	<0.001	7200 (99.1)	2973 (99.3)	4227 (99.0)	0.23	5400 (95.6)	2022 (94.9)	3378 (96.1)	0.03
Visitor	82 (1.4)	16 (0.7)	66 (1.8)		65 (0.9)	22 (0.7)	43 (1.0)		246 (4.4)	109 (5.1)	137 (3.9)	
Missing	3 (0.1)	0	3		4 (0.1)	0	4		0	0	0	
**Pregnancy < = 50yrs**												
Yes	338 (9.9)		338 (9.9)		257 (6.6)		257 (6.6)		130 (4.3)		130 (4.3)	
No	3023 (88.2)		3023 (88.2)		3627 (92.8)		3627 (93.0)		2858 (93.9)		2858 (94.0)	
Don’t know	68 (2.0)		68(2.0)		17 (0.4)		17 (0.4)		53 (1.7)		53 (1.7)	
Missing	0		0		8 (0.2)		8		2 (0.1)		2	
**Currently breastfeeding**												
Yes	1095 (52.6)		1095 (52.9)		1197 (55.2)		1197 (55.5)		307 (31.2)		307 (31.2)	
No	976 (46.9)		976 (47.1)		961 (44.4)		961 (44.5)		677 (68.8)		677 (68.8)	
Missing	12 (0.6)		12		9 (0.4)		9		0			

**Table 2:** Showing age, CD4 cell count and eligibility distribution by sex among 4,113 HIV-positive individuals.

Country	Kenya				Malawi				South Africa			
Variable	N(%)	Male n(%)	Female n(%)	P value	N(%)	Male n(%)	Female n(%)	P value	N(%)	Male n(%)	Female n(%)	P value
Total	1457	457	1000	-	1233	394	839	-	1423	338	1085	-
**Age group**												
15–19	62 (4.3)	7 (1.7)	55 (5.2)	<0.001	28 (2.3)	7 (1.6)	21 (2.3)	0.002	70 (4.9)	12 (3.1)	58 (5.2)	0.06
20–34	701 (48.1)	158 (33.7)	543 (54.4)		470 (38.1)	116 (30.4)	354 (41.6)		677 (47.6)	140 (42.0)	537 (49.3)	
35–44	284 (26.4)	152 (32.6)	232 (23.4)		452 (36.7)	165 (41.4)	287 (34.6)		386 (27.1)	105 (30.0)	281 (26.7)	
45–59	310 (21.3)	140 (31.9)	170 (17.0)		283 (23.0)	106 (26.6)	177 (21.6)		290 (20.4)	81 (25.0)	209 (18.8)	
**CD4 cell count**												
< = 350	419 (28.8)	165 (39.5)	254 (28.1)	<0.001	336 (27.3)	151 (40.8)	185 (22.8)	<0.001	387 (27.2)	141 (44.3)	246 (23.1)	<0.001
351–500	342 (23.5)	115 (26.8)	227 (23.0)		300 (24.3)	95 (25.7)	205 (24.9)		362 (25.4)	81 (23.5)	281 (26.2)	
501+	615 (42.2)	146 (33.7)	269 (49.0)		545 (44.2)	122 (33.4)	423 (52.4)		651 (45.8)	110 (32.2)	541 (50.7)	
Missing	81 (5.6)	31	50		52 (4.2)	26	26		23 (1.6)	6	17	
**Eligibility**												
Ever ART	587 (40.3)	189 (44.0)	398 (40.8)	0.01	653 (53.0)	208 (55.4)	445 (54.2)	-	758 (53.3)	175 (50.6)	583 (54.5)	-
CD4< = 350	242 (16.6)	91 (21.1)	151 (17.1)		153 (12.4)	77 (20.7)	76 (9.0)		188 (13.2)	75 (25.0)	113 (10.6)	
Pregnant + CD4>350	-	-	-		21 (1.7)	-	21 (2.5)		37 (2.6)	-	37 (3.3)	
Breastfeeding + CD4>350	-	-	-		132 (10.7)	-	132 (16.3)		44 (3.1)	-	44 (4.2)	
CD4 351–500	178 (12.2)	57 (13.9)	212 (12.4)		89 (7.2)	41 (11.1)	48 (5.9)		146 (10.3)	34 (10.0)	112 (10.4)	
CD4 501+	373 (25.6)	91 (21.0)	282 (29.8)		142 (11.5)	47 (12.7)	95 (12.1)		231 (16.2)	50 (14.5)	181 (16.9)	
Missing	77 (5.3)	29	48		43 (3.5)	21	22		19 (1.3)	4	15	

Table 3 shows changes in proportion of people who will become eligible for ART with changes in eligibility criteria. Overall 2,816 (70.8%) were classified as “eligible for ART” based on either ever having started ART or meeting the country eligibility criteria for ART initiation. Individual country proportions of patients eligible for ART were 60.0% (95% CI: 57.2–62.7) in Kenya, 73.4% (70.8–75.8) in South Africa and 80.1% (77.3–82.6) in Malawi. Applying the CD4< = 500 guidelines, Kenya would increase the proportion eligible for ART from 60% to 82.0% (79.8–84.1) (a 36% increase in the proportion eligible) and a further 18.0% of the population that would become eligible in a “test and treat” scenario. In South Africa shifting from 2011 national ART guidelines to CD4< = 500, 83.7% (81.5–85.6) of South African would become eligible for ART (a 14% increase in the proportion eligible) and a further 16.3% would be eligible in a “test and treat” scenario. In Malawi moving from 2011 national ART guidelines to CD4< = 500 only 7.4% more would become eligible as about 87.6% (85.0–89.8) would become eligible and a further 12.4% would be eligible by implementing “test and treat”. Due to Kenya recommending Option A for PMTCT at the time of the survey, implementation of CD4< = 500 guidelines would increase eligibility for women more than men. In contrast, Malawi that had implemented Option B+ for PMTCT at the time of the survey, introduction of CD4< = 500 guidelines would increase eligibility for men more than for women while for South Africa that had implemented option B for PMTCT at the time of the survey, adopting CD4< = 500 guidelines would have similar effect between men and women. Among 746 individuals that would become eligible for ART as a result of “test and treat”, 74.8% were women and 6.7% were individuals aged 15–19 years. We found that men and women were affected equally in all three countries moving from CD4< = 500 to “test and treat”. In all countries, those aged 15–19 years would experience the biggest change in eligibility comparing CD4< = 500 and “test and treat” guidelines with about 20% of patients becoming eligible. By sex, country specific results among adolescents, we found that 21.3% female and 70.6% male in Kenya, 26.1% female and 20.0% male in Malawi and 16.3% female and 34.2% male in South Africa would become eligible moving from CD4< = 500 to “test and treat” however in all countries the number of males were very few.

ART coverage (of those eligible in terms of country ART initiation guidelines at the time of the surveys) was highest in Malawi. 82.0% (79.0–84.6) of all HIV-positive individuals eligible for ART were receiving treatment while ART coverage in South Africa and Kenya was almost the same 71.8% (68.6–74.8) in South Africa and 71.3% (67.3–74.9) in Kenya.

**Table 3:** Showing percentage change in eligibility between ART eligibility criteria at the time of the survey 2012 and 2013 WHO art guidelines.

Country	Kenya			Malawi			South Africa		
Variable	Kenya 2011 national ART guidelines	WHO 2013	% Diff. with WHO 2015	Malawi 2011 national ART guidelines	WHO 2013	% Diff. with WHO 2015	South Africa 2013 national ART guidelines	WHO 2013	% Diff. with WHO 2015
Overall	60.0 (57.2–62.7)	82.0 (79.8–84.1)	18.0 (15.9–20.2)	80.1 (77.3–82.6)	87.6 (85.0–89.8)	12.4 (10.2–15.1)	73.4 (70.8–75.8)	83.7 (81.5–85.6)	16.3 (14.4–18.5)
**Gender**									
Male	65.1 (60.3–69.7)	79.1 (74.7–82.8)	21.0 (17.2–25.3)	76.2 (71.5–80.2)	87.3 (83.2–90.5)	12.7 (9.5–16.8)	75.5 (70.2–80.2)	85.5 (81.4–88.9)	14.5 (11.2–18.6)
Female	57.7 (54.6–60.8)	83.4 (80.8–85.6)	16.6 (14.4–19.2)	81.8 (78.6–84.6)	87.7 (84.7–90.2)	12.3(9.8–15.3)	72.7 (69.8–75.4)	83.1 (80.6–85.3)	16.9 (14.7–19.4)
**Age group**									
15–19 yrs	25.9 (14.9–41.1)	72.2 (59.8–82.0)	27.8 (18.1–40.2)	71.2 (48.7986.5)	75.5 (52.7–89.5)	24.5 (10.5–47.3)	65.6 (54.3–75.4)	80.8 (69.3–88.7)	19.2 (11.3–30.7)
20–34 yrs	51.4 (47.0–55.8)	80.1 (76.4–83.3)	19.9 (16.7–23.6)	75.5 (70.9–79.6)	83.2 (79.1–86.6)	16.8 (13.4–20.9)	65.3 (60.7–69.5)	79.4 (75.4–83.0)	20.6 (17.0–24.7)
35–44 yrs	67.6 (62.5–72.3)	83.8 (80.1–87.0)	16.2 (13.0–19.9)	83.3 (79.0–86.9)	91.7 (88.2–94.3)	8.3 (5.7–11.8)	82.6 (77.4–86.9)	88.3 (83.4–91.8)	11.8 (8.2–16.6)
45–59 yrs	75.1 (70.0–79.7)	85.9 (80.8–89.8)	14.1 (10.2–19.2)	82.8 (76.0–88.0)	88.8 (82.2–93.2)	11.2 (6.8–17.8)	81.6 (76.5–85.8)	88.1 (83.1–91.7)	11.9 (8.3–16.9)

**Fig 2:**
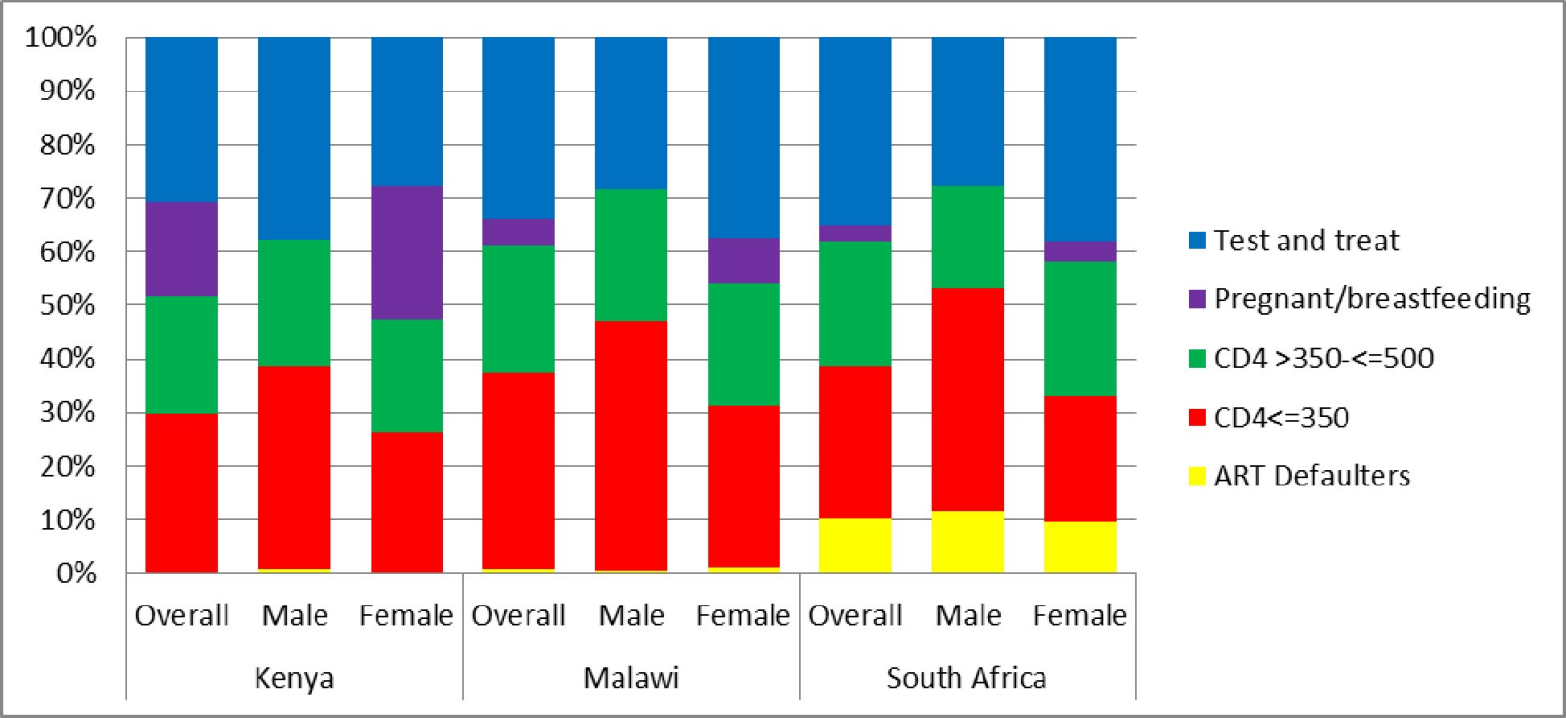
Categories of ART eligibility among individuals not on ART. Categories of ART eligibility in which individuals currently not on ART fall based on different ART eligibility guidelines, overall and by sex for each country.

Given the stricter criteria for ART initiation and lower coverage, Kenya had the highest proportion of HIV-positive individuals who were not on ART; 808 (58.0%), followed by South Africa 655 (46.9%) and Malawi 429 (35.7%). Of these, the proportion of individuals with CD4 of < = 350 was no more than 36% and similar across countries. Similarly, the proportion of those not on ART with CD4 between 350 and 500 was similar across countries and was no more than 25% for each country. Overall, of the HIV-positive individuals not on ART, the proportion who would be started on ART only because of test and treat (i.e. not pregnant or breast-feeding and have CD4> 500) ranged from 28.4 (24.4–32.5) in Kenya to 37.9% (33.4–42.7) South Africa and 39.7% (32.1–47.8) Malawi among females while it ranged from 26.3% (20.3–33.3) in South Africa to 29.9% (22.8–38.2) Malawi and 35.7% (29.3–42.7) Kenya among males Fig 2.

## Discussion

Our results have shown that at least 30% more HIV-positive individuals (CD4>500 cells/μl) not on ART would become eligible for ART as a result of “test and treat” policy. The biggest proportionate change occurred in those 15–19 years of age. The transition to “test and treat” would, however, be much easier for countries that have already implemented the CD4< = 500 guidelines and have high ART coverage.

In addition to the benefits of ART in lowering mortality and morbidity [6–8] and of starting treatment early [12, 23, 24], achieving low viral load among HIV-positive individuals has potential benefits in reducing HIV transmission [9–12]. One way of achieving lower viral load in a population is to increase ART coverage. Although some people would argue that increasing eligibility will only have a big impact in countries where ART coverage is already high, increasing eligibility especially by test and treat will itself help improve coverage especially in countries where failure to complete ART eligibility assessment is one of the factors contributing to low ART coverage as some studies have shown [25]. Our findings regarding eligibility in terms of different guidelines are comparable to other studies. The Kenya AIDS Indicator Survey (KAIS) 2012 [16] which was a nationally representative survey estimated that 58.8% of HIV-positive individuals were eligible for ART according to Kenya National ART 2011 guidelines and about 18.6% more people would be eligible if Kenya were to adopt the CD4< = 500 guidelines, which translates to an additional 22.6% more individuals if Kenya were to move to “test and treat” compared to the 18.0% we found. The small difference however could be partly explained by differences in age groups for which estimates were calculated. KAIS used 15–64 years while we used 15–59 years.

While our findings show no major differences in the change in proportions eligible by sex across all the three countries if eligibility criteria are changed to treating all, there was a noticeable trend by age group. Our results show that a consistently higher proportion of adolescents (in comparison to adults) in all three countries would be in need of ART by adopting the “test and treat” guidelines. This is not surprising as adolescents are more likely to be recently infected and thus have high CD4 values, and are less likely to be aware of their diagnosis. Adolescents and adults face different challenges in accessing ART [26]. Many adolescents face stigma and discrimination from their peers [27, 28]. They may find it difficult to access treatment if hospital visits coincide with school or if they are in boarding schools, or if the clinic is far and they cannot easily manage to pay for transport costs [27]. In some countries, the need for parental consent for HIV testing and treatment in those <18 years of age may be a barrier to accessing HIV care [29, 30]. Adolescents are also undergoing rapid developmental changes especially neurocognitive changes that may make it particularly difficult to remain in care and adhere to treatment [31–33]. This age group therefore, will need different social support structures and, programs that are youth friendly to help them access and adhere to the treatment [4, 34, 35]. Without proper support structure among adolescents, there is a high risk of poor adherence with development of drug resistant HIV strains. However, the benefits of increased numbers of people on ART from a young age such as reducing number of new horizontal and vertical infections and deaths greatly outweighs the increased drug resistance that will be generated[36].

Although most countries have adopted the “test and treat” policy, for some countries, implementation has not moved at a fast pace as only 55 (40%) of low and middle income countries (LMIC) have put the policy for Treat All fully into practice and 12 (9%) LMIC have implemented treat all in a majority of treatment sites [37] perhaps due to the cost associated with increased number of individuals on ART. However, the costs and benefit analyses that have been done on increasing eligibility from CD4 350 to 500 or to all HIV-positive individuals using different models found encouraging results. Those studies found relatively low cost of expanding ART to individuals with CD4 < = 500 or everyone who is positive compared to the benefits that would be accrued when many people are started on ART [38–40]. However, they concluded that for the test and treat policy to have bearing positive effects on populations, it has to be accompanied by increased coverage of ART although this does not guarantee increased number of people on ART as a certain proportion of those eligible will either remain undiagnosed unless testing is expanded, or will choose not to initiate ART. As all the three countries included in this analysis have now implemented test and treat, it is encouraging to see significant increases in ART coverage from the year just before implementation of test and treat to the year after, 61.0% (2015) vs 75.0% (2017) in Kenya, 60.0% (2015) vs 71.0% (2017) in Malawi and 52.0% (2015) vs 61.0% (2017) in South Africa [41] although more research need to be done to attribute this increase to implementation of new guidelines.

Our study may be biased due to lack of objective measures on certain important variables used to determine ART eligibility, for example whether individuals were on ART or not relied on self-reported information (Kenya and Malawi), as well as self-report of pregnancy and breastfeeding (all three countries). However, risk of individuals giving inaccurate information regarding ART use was reduced as participants were asked to show their health books or pills if they said they were on ART and the agreement between ART Laboratory tested and self-report in the South African study was found to be generally good [42]. Also, the lack of clinical information to classify eligibility by WHO clinical staging, TB or hepatitis B virus co-infection with AIDS and being in a serodiscordant couple may have underestimated the number of ART eligible individuals at the time of the survey. In addition, among the people who participated, only a small proportion had missing information in one or more of the following variables: HIV awareness, viral load, education, marital status, occupation, residency status, pregnancy and breastfeeding information. Fewer than 4.0% of HIV-positive individuals were missing information on CD4, hence assessment of eligibility in terms of CD4 was near complete.

One of the main advantages of this study was the ability to have three large studies put together which allows for comparison of different country experiences at different stages of implementing the WHO guidelines and different stages of the HIV epidemic which gave us the opportunity to compare how much impact moving from one guideline to another would have on countries.

Since all the three countries in this study have now moved to test and treat. This decision is supported by our study results–for those countries that had already implemented option B, or B+, the proportion of additional individuals to be started on ART would be relatively small, with potential benefits of reduced morbidity, mortality and transmission of moving to test and treat. For Kenya where eligibility and coverage were low, test and treat offers the opportunity to promote increased coverage. To achieve the benefits of test and treat, specific support for the groups where “test and treat” has the most impact on eligibility, particularly adolescents, is needed. We therefore, need to continue to evaluate programs to assess the fidelity of implementation of “test and treat” guidelines and whether the promised benefits are being achieved.
